# Dietary niche shapes bacterial community in Indo-Pacific ants

**DOI:** 10.1128/spectrum.01965-25

**Published:** 2025-09-03

**Authors:** Phoebe Cunningham, Rebecca Vesey, Hannah Chaudry-Phipps, Tom M. Fayle, Umar Diara, Guillaume Chomicki, Dan Lestina, Jiri Tuma, Pamela H. Templer, Petr Klimes, Lee M. Henry

**Affiliations:** 1 School of Biological and Behavioural Sciences, Queen Mary University of London, London, United Kingdom; 2 Biology Centre of the Czech Academy of Sciences, Institute of Entomology, Ceske Budejovice, Czech Republic; 3 School of Agriculture, Geography, Environment, Ocean and Natural Sciences, The University of the South Pacific, Suva, Fiji; 4 Department of Bioscience, Durham University 3057, Durham, United Kingdom; 5 Faculty of Science, University of South Bohemia 204738 , Ceske Budejovice, Czechia; 6 Biology Centre of the Czech Academy of Sciences, Institute of Soil Biology and Biogeochemistry, Ceske Budejovice, Czech Republic; 7 Department of Biology, Boston University 168346, Boston, Massachusetts, USA; Cleveland Clinic Lerner Research Institute, Cleveland, Ohio, USA

**Keywords:** microbiome, 16S rRNA sequencing, symbioses, tropics, diet evolution

## Abstract

Ants are among the most ecologically diverse insects, especially in tropical forest ecosystems, yet what shapes their microbial associates remains poorly understood. Most research has focused on Neotropical ants, where strong microbial associations have been linked to shifts in diet—such as herbivory—and nesting ecology. In contrast, Indo-Pacific ants, which have independently evolved similar specialized lifestyles, remain largely unstudied for their microbial associations. Here, we integrate deep-coverage 16S rRNA sequencing with stable isotope (δ^15^N) analyses to characterize the microbiomes of 239 ants from 36 species across 24 genera from the Indo-Pacific region. These ants span a broad ecological range, from ground-dwelling predators to strict arboreal herbivores, allowing us to ask: which ecological traits shape bacterial diversity in tropical ants? Our results reveal that dietary niche (δ^15^N)—rather than nesting habitat—is the primary explanatory variable of microbial diversity and composition. Ants consuming protein-rich diets (predators and omnivores) exhibit significantly higher bacterial alpha diversity and distinct community composition profiles compared to their plant-based (herbivorous) counterparts, with Rickettsiales, Rhizobiales, and Enterobacterales as major contributors to these differences. Notably, herbivorous ants tend to harbor simpler, Enterobacterales-dominated microbiomes, whereas predators support more complex communities, frequently containing Rhizobiales bacteria. Phylogenetic comparative analyses suggest that the acquisition of specific bacteria, particularly Enterobacterales, is strongly correlated with evolutionary transitions toward herbivory. Our findings expand on previously documented trends in ant-microbe interactions while also offering new insights into how symbioses drive the evolution of dietary strategies in tropical ecosystems.

**IMPORTANCE:**

Host-microbe interactions have played an integral role in the evolution of specialized lifestyles in insects. Ants, with their ecological diversity and broad microbial associations, offer a powerful model for studying these dynamics. However, most research has focused on Neotropical ant lineages, limiting our broader understanding of how microbes influence ant evolution. Our study addresses this gap by examining Indo-Pacific ants—an underexplored but ecologically rich group—and reveals that diet, rather than nesting habitat, is the primary driver of microbial diversity. Notably, our findings challenge established patterns: Rhizobiales are more frequently associated with predatory ants than herbivores, contrasting with trends in Neotropical taxa. Furthermore, phylogenetic analyses suggest Enterobacterales may have played a key role in the evolution of herbivory. These results underscore the value of expanding research beyond taxa in well-studied regions and show how microbial partnerships can both reinforce and reshape our understanding of lifestyle evolution in ants.

## INTRODUCTION

Bacterial symbioses have shaped the distribution of life on Earth, driving niche expansions from the colonization of nutrient-poor soils in plants to deep-sea communities (1, 2). Insects, in particular, have formed relationships with diverse symbiotic microbes that have facilitated radiations into previously uninhabitable niches that have shaped their diversification (3). From exclusive sap- and blood-feeding to cuticle fortification and defense against natural enemies, insects display a myriad of adaptations that are afforded by symbiont acquisition (4–6).

Ants are among the most ecologically diverse and abundant organisms, thriving across diverse habitats (7). Like many insects, ants form strong microbial associations, from single bacterial symbionts housed in specialized cells called bacteriocytes, to heritable reproductive parasites and complex gut communities (8–11). The evolution of stable bacterial symbioses in ants is often associated with specialized diets such as strict herbivory or predation (11, 12). For example, several ancestrally predatory lineages have evolved bacterial symbioses that enabled survival on low-protein, carbohydrate-rich diets like honeydew from sap-feeding insects and extrafloral nectaries (13). *Cephalotes* turtle ants harbor stable gut microbiomes that recycle nitrogen from urea, while Camponotini ants host the bacteriocyte-associated symbiont *Blochmannia* that provides essential amino acids to support a diet primarily of plant-derived resources, such as insect honeydew and plant nectar (14–20). Even predatory ants like *Harpegnathos saltator* possess gut-associated symbionts such as *Bartonella*, capable of amino acid synthesis, although their exact role remains unclear (21). However, most of what we know about ant–microbe interactions comes from studies on Neotropical ant lineages, limiting our broader understanding of the factors shaping bacterial-ant associates.

Research on Neotropical ant microbiomes has uncovered several interesting trends. Besides *Cephalotes*, significant focus has been placed on *Dolichoderus*, Neotropical army ants, and attine fungus-farming ants (10, 22–24). Army ants in the genera *Eciton*, *Labidus,* and *Nomamyrmex* harbor specialized gut bacteria that potentially aid in pathogen defense and nutrient provisioning (25). Similarly, *Attine* leaf-cutter ants are associated with Mollicutes and Rhizobiales gut bacteria, presumably to aid in nutrient uptake, and cuticular *Actinobacteria* that produce antibiotics to protect against fungal pathogens such as *Escovopsis* (23, 26, 27). Herbivorous *Dolichoderus* and predaceous *Daceton armigerum* have both formed strong associations with Rhizobiales bacteria (12, 24, 28). In contrast, omnivorous ants such as *Azteca* and Neotropical *Crematogaster* harbor surprisingly few bacteria (10). Whereas both diet and nesting habitat have been shown to influence bacterial composition in Amazonian ants (29). These patterns suggest microbial acquisitions can be influenced by both feeding and nesting habitats in ants. However, our understanding of how ant ecology shapes bacterial diversity and stability, and the role of bacterial acquisitions (e.g., Rhizobiales) on niche shifts in ants, remains limited.

Indo-Pacific ants have also evolved specialized feeding strategies, including strict predation, herbivory, and fungus-farming, but how these ecological traits have influenced their microbial associations remains poorly studied. Existing research suggests that ant ecology may influence host bacterial diversity in predictable ways. For example, honeydew-feeding *Tetraponera* ants evolved a specialized gut pouch containing bacteria related to plant-associated nitrogen fixers (30, 31). Predatory weaver ants (*Oecophylla smaragdina*) harbor sugar- and acid-tolerant bacteria (Acetobacteraceae, Lactobacillaceae) possibly linked to their acidic defensive sprays (30). Similar bacteria also occur in the predatory Neotropical Argentine ant (*Linepithema humile*), suggesting bacterial community convergence related to defensive lifestyles (30, 31). Ant-plant mutualisms, where myrmecophytes host ant colonies, occur globally in the tropics (e.g., *Azteca*, *Anonychomyrma*, *Podomyrma*, *Pseudomyrmex*, *Philidris*, and *Cataulacus*), often involving plant-derived diets, but research on their gut microbes is limited (32–37). Overall, a paucity of research into species from understudied regions such as the Indo-Pacific has constrained our understanding of how host ecology shapes bacterial diversity and whether ants that have converged on similar ecological niches have also converged on similar microbial partnerships.

Here, we use deep-coverage 16S rRNA sequencing to test whether feeding niche and nesting habitat influence bacterial diversity in 36 ant species from 24 genera across the Indo-Pacific region, spanning lifestyles from strict predators to herbivorous ant-plant mutualists. Natural abundance stable nitrogen isotope analysis is an effective tool for characterizing the trophic level in ants (38–40). By combining new and published natural abundance stable isotope data and information on diet and nesting habitat, we examine bacterial community-ant lifestyle relationships. Using this data, along with a host phylogeny developed for this study, we asked three key questions: (i) what best explains bacterial diversity in ants, the feeding niche, or nesting habitat? and (ii) are the same bacterial associations found in ants that have convergently evolved similar lifestyles? and (iii) has the acquisition of certain microbes repeatedly accompanied evolutionary transitions to specialized lifestyles?

## RESULTS

**Fig 1:**
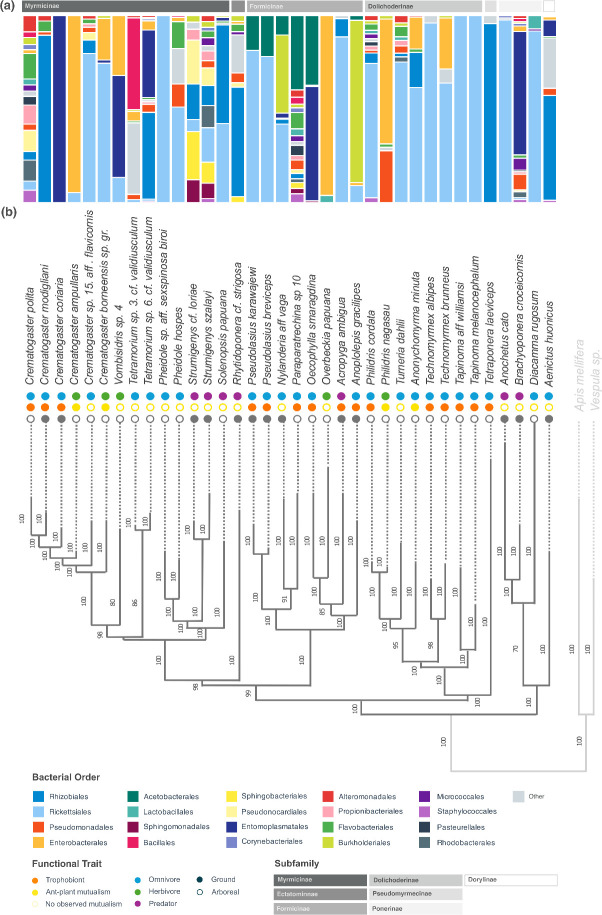
Taxonomic bar plots showing bacterial community diversity of Indo-Pacific ant lineages. (**a**) Taxonomic bar plot showing average relative abundances of bacterial orders for each species shown in the ant phylogeny and (**b**) host phylogeny of study species generated from two housekeeping genes, Cytochrome Oxidase I and Wingless, see text for details. Circles at branch tips represent ecological traits, and numbers on branches represent bootstrap support. Outgroups are shown in light gray. Bootstrap support values were added only at nodes without polytomies (see Fig. S1).

In total, we analyzed the 16S rRNA gene in 239 ants (168 adult workers, 47 larvae, 16 pupae, and 8 queens) from 36 species, 24 genera, and 7 subfamilies, which include multiple independent evolutionary transitions into different feeding niches (Fig. 1; Table S1). Based on δ^15^N thresholds (where <2 herbivore; 2–6 omnivore; >6 predator), our data set contained 5 herbivorous, 24 omnivorous, and 7 predaceous species, as well as 4 ant-plant mutualists, 15 trophobiont-tending mutualists, and 17 species presenting no observed mutualism with plants or trophobionts. All species information, including sample sizes per species, can be found in Table S2. Sequencing generated a total of 8,801,029 reads, with a mean of 32,140 reads per sample (Table S3). Rickettsiales was the most abundant bacterial order, accounting for >25% of reads across all samples. This was followed by Rhizobiales (~8%), Pseudomonadales (~8%), Enterobacterales (~6%), Flavobacteriales (~6%), and Burkholderiales (~6%), in order of abundance.

### Ant ecological traits influencing bacterial community diversity and composition

**Fig 2:**
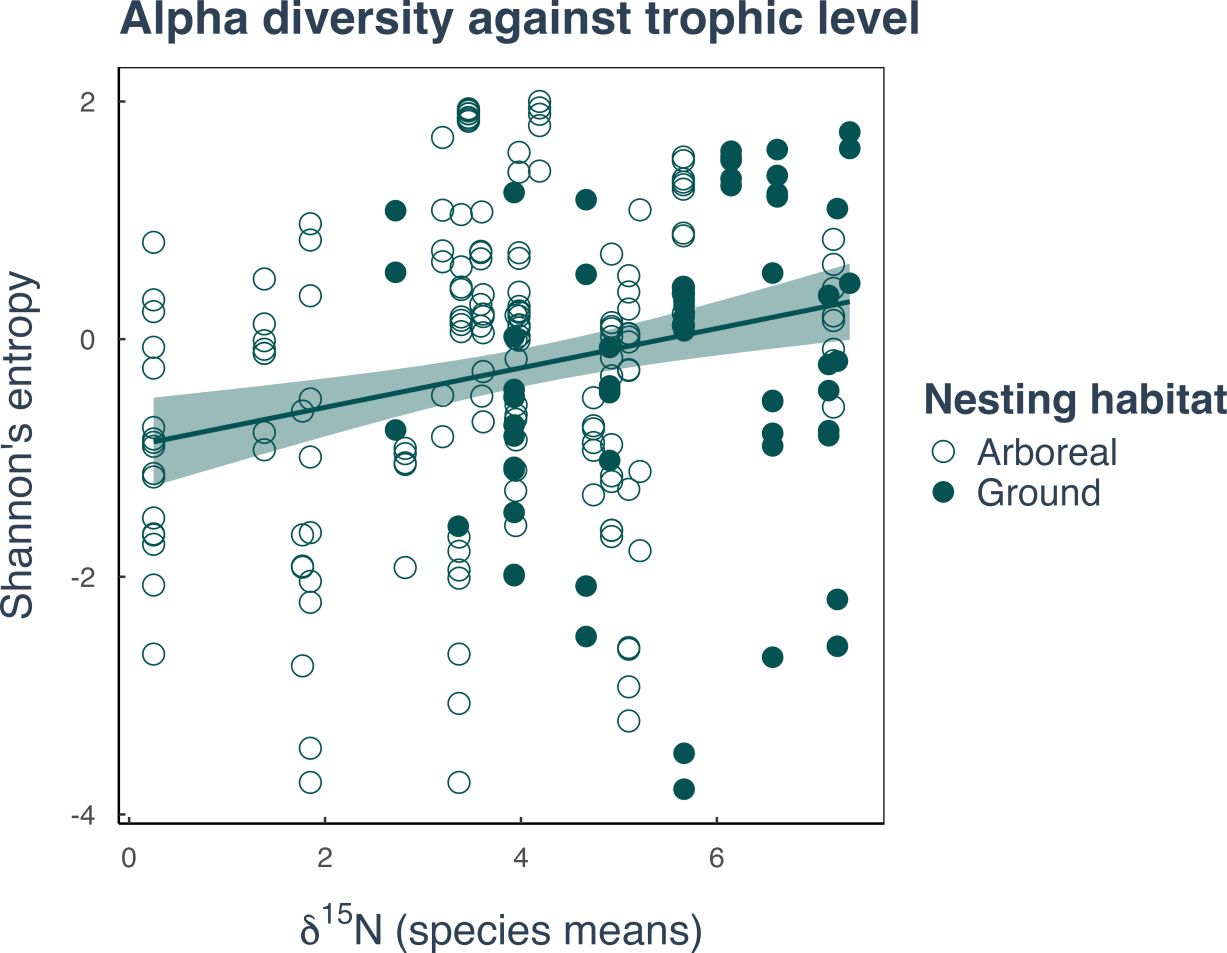
Correlation between bacterial alpha diversity with ant diet and nesting habitat. Alpha diversity represented by Shannon’s entropy shows a positive correlation with the mean δ^15^N score per species as an indication of the level of dietary protein for ground nesting (filled) and arboreal (open) nesting habitats. Statistics were generated from MCMCglmm models (Table S5).

**Fig 3:**
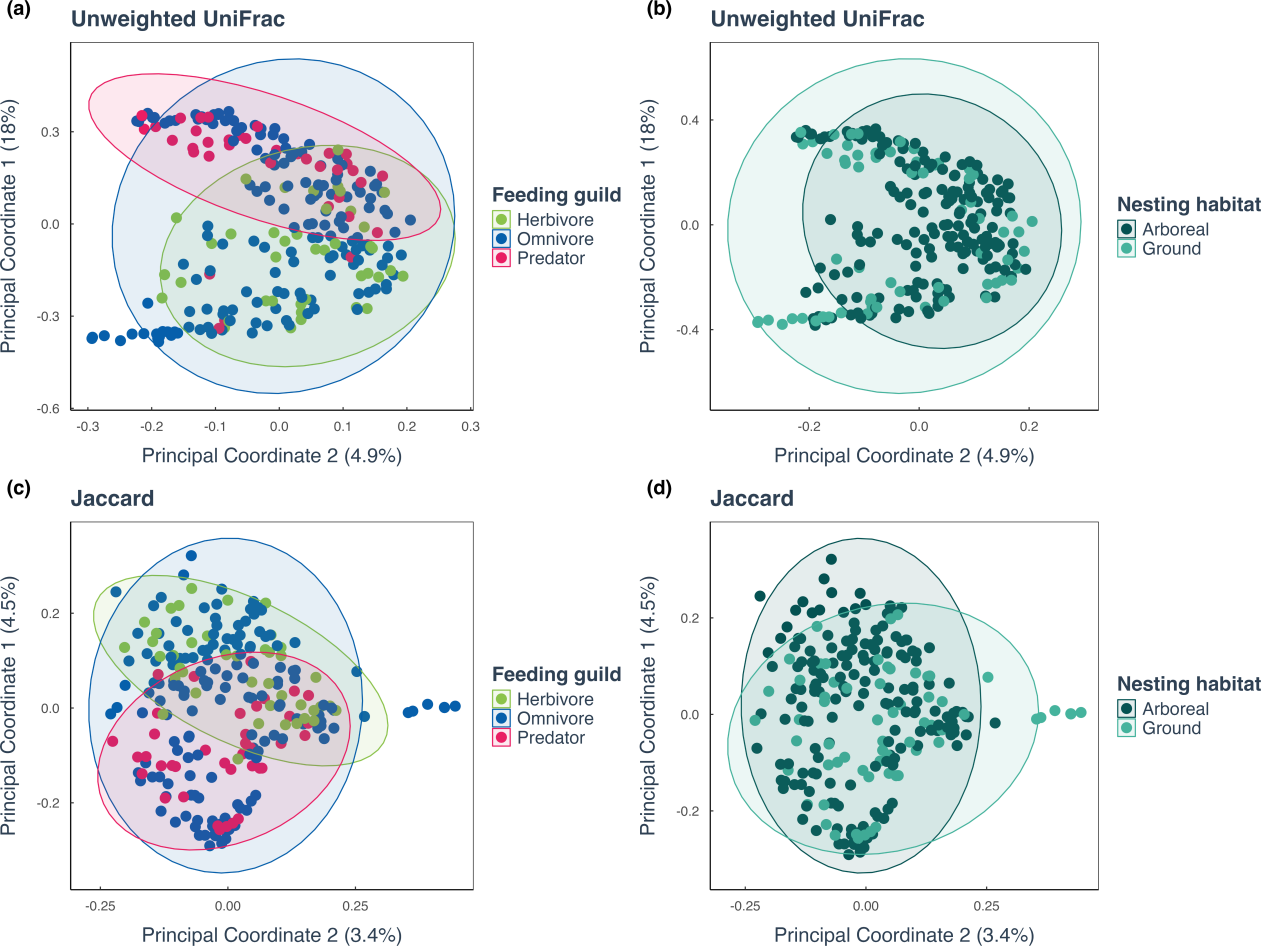
Bacterial community composition with feeding guild and nesting habitat. Community composition, measured by the unweighted UniFrac index, is colored by (**a**) feeding guild (herbivores in green, omnivores in blue, predators in pink) and (**b**) nesting habitat (dark green for arboreal, light green for ground habitats). The same ecological traits are shown with Jaccard’s index in (**c**) and (**d**). See Tables S5 and S6 for MCMCglmm model outputs and Fig. S4 principal coordinate analysis (PCoA) plots for Bray-Curtis and weighted UniFrac.

We found that diet (measured using δ^15^N stable isotope score) was the most significant factor in predicting the alpha diversity of a species bacterial community (Fig. 2; Fig. S3). Using MCMCglmm models, including host phylogeny as a random effect, we found that species with a higher δ^15^N score, and therefore a more proteinaceous diet, tended to have a greater variety of bacterial species present. This was illustrated by a posterior mean and credible intervals above zero for both Shannon’s entropy and Faith’s PD, with more than 99% of the estimates above zero for both (Shannon β = 0.32, 95% CI: 0.15–0.50, >99% of posterior >0; Fig. 3; Faith’s PD β = 0.20, 95% CI: 0.08–0.32, >99% of posterior >0; Table S6; Fig. S3). We did not find any support that nesting habitat, measured as arboreal or ground-nesting, impacts the bacterial diversity of species in this data set (Table S6).

We found that species with a more proteinaceous diet also had a significantly different composition of bacteria compared to species with a lower protein diet (Fig. 3a and c). MCMCglmm models of the first principal coordinate (PC1) from each diversity metric revealed that two out of four metrics were significantly correlated with diet, as indicated by δ^15^N values (Jaccard β = −0.04, 95% CI: −0.06 to −0.02; <1% of posterior >0; unweighted unifrac; β = 0.05, 95% CI: 0.02–0.09; >99% of posterior >0; Table S7; Fig. 3a and c). Bray-Curtis and weighted UniFrac metrics were not significant (Table S7; Fig. S4a through d). Both Jaccard and unweighted UniFrac metrics do not account for abundance, suggesting that the presence and absence of bacterial species, rather than their relative amounts, was the primary factor contributing to this trend. In contrast, nesting habitats, arboreal vs ground-nesting, did not have an influence on ant bacterial community composition (Table S7; Fig. 3b and d).

### Bacterial orders contributing the most to the variance observed

**Fig 4:**
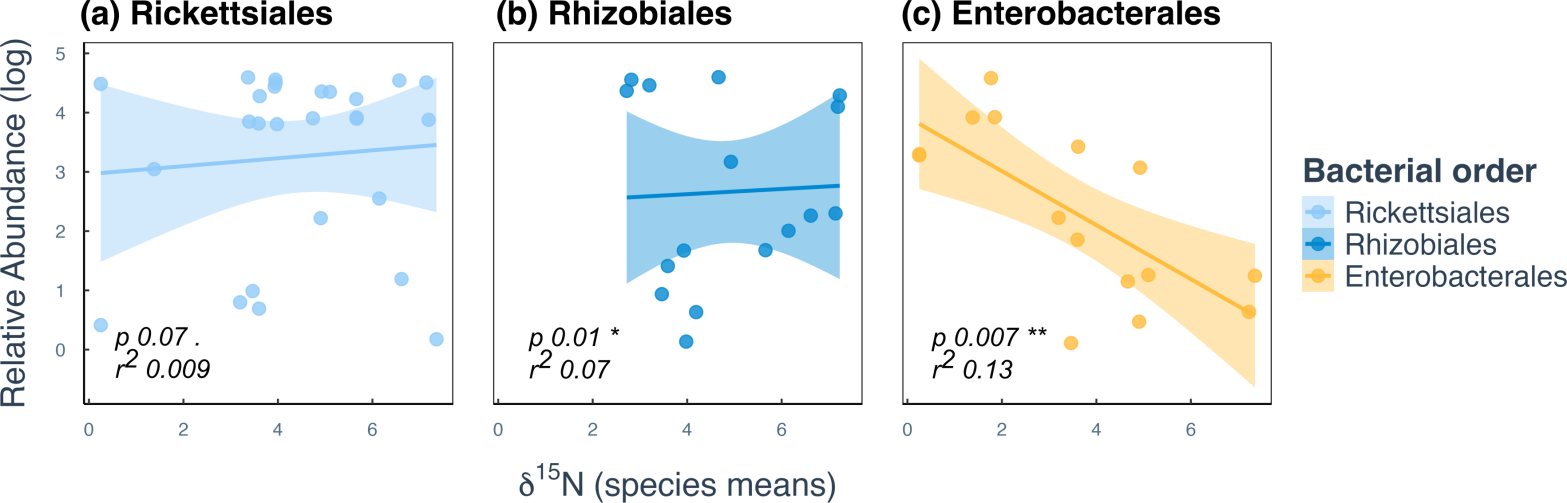
Correlation between diet (δ^15^N) and the relative abundance of the top three bacterial orders driving ant bacterial diversity. Linear regressions were run on all samples for (**a**) Rickettsiales (*P* 0.07, r^2^ 0.009); (**b**) Rhizobiales (*P* 0.01*, r^2^ 0.07), and (**c**) Enterobacterales (*P* 0.007**, r^2^ 0.13), identified via SIMPER as the main contributors to variance (Table S8). Points represent species means for visualization, but *P* values are based on linear regression of all samples.

Using SIMPER tests to examine the contribution of specific bacterial orders to the dissimilarity observed, we identified Rickettsiales, Rhizobiales, and Enterobacterales as the top three contributors to bacterial community variation across feeding groups (Table S8). Linear modeling showed Enterobacterales were negatively correlated with a protein-rich diet, whereas Rhizobiales were positively correlated with a protein-rich diet (and were absent in herbivores), and Rickettsiales was relatively consistent among feeding groups (Fig. 4). There was no relationship between ant diet and the abundance of the fourth, fifth, and sixth most prevalent orders, Pseudomonadales, Flavobacteriales, and Burkholderiales, but notably, all three of these bacterial orders were absent in strict herbivores (low δ^15^N scores). This suggests herbivores in our study have simpler gut microbiomes dominated by one or two highly abundant bacterial partners, often Enterobacterales, whereas predators and omnivores exhibit more diverse microbiomes with multiple microbial partners. This is reflected in alpha diversity, with predators harboring the highest bacterial diversity: 232 OTUs across 37 samples compared to only 72 OTUs in 32 herbivore samples (Fig. S2a).

Rickettsiales was the most prevalent bacterial order, found in 76% of samples and comprising 26% of all OTUs: most of these reads—nearly 99%—belonged to the genus *Wolbachia*. It also contributed the most to dissimilarity between feeding groups: 43% between herbivores and omnivores, 32% between herbivores and predators, and 45% between omnivores and predators, driven by differences in prevalence and relative abundance (Table S8). Interestingly, although *Wolbachia* was present in more than 68% of the samples across feeding groups, it represented a much larger proportion of the OTUs in herbivores: 39% of OTUs, compared to 26% in omnivores and 18% in predators (Table S9). This likely reflects the lower bacterial community diversity in herbivores—when *Wolbachia* is present, it often dominates, whereas in omnivores and predators, it coexists with a more diverse bacterial community (Table S6; Fig. S2a).

Rhizobiales was the second most prevalent order among all the OTUs, found in 19% of samples and representing 8% of all OTUs. This order also contributed strongly to the dissimilarity between feeding groups, most noteworthy being its complete absence in strictly herbivorous species (δ^15^N < 2) in our data set (Fig. 4). Omnivores also have a relatively low prevalence of Rhizobiales, present in only 15% of samples and 6% of the total omnivore OTUs. By comparison, Rhizobiales is present in 51% of samples and represents 17% of all OTUs in predators. Omnivores tended to have the most taxonomic variation within the bacterial order, with representation across eight different genera of Rhizobiales, compared to six in predators, despite predators having a greater proportion of their reads assigned to Rhizobiales. Of the reads that could be assigned to species level, two of the Rhizobiales OTUs were assigned to *Candidatus Tokpelaia* in the army ant *Aenictus huonicus*. The genus *Bartonella* was also found to be highly abundant in the omnivorous *Tetraponera laeviceps* and *Crematogaster polita* (Fig. S2a), and in moderate abundance in *C. modigliani* and predaceous *Strumigenys szalayi*. We also found a strong association between Rhizobiales and the predatory ant *Rhytidoponera* cf. *strigosa*. The same OTU was found at over 90% abundance in the three workers sequenced and was not able to be identified beyond the family level, suggesting some divergence from free-living relatives. A strong association with Rhizobiales was also observed in *Tetramorium* sp. 6 cf. *validiusculum* that was related to a previously identified uncultured *Rhizobiales* (Table S3).

Pseudomonadales was the third most abundant bacterial order in our data set; however, it contributed little to differences between ant feeding guilds. Its relatively high abundance was due to it being prevalent in *Philidris nagasau*, where Acinetobacter dominated in 3 of 10 workers (Fig. S2a). Acinetobacter was the most common Pseudomonadales genus, occurring across all feeding guilds, along with *Enhydrobacter* and *Pseudomonas*.

Enterobacterales was the fourth most prevalent order, but contributed substantially to observed variance. It comprised nearly 33% of herbivore OTUs and appeared in 50% of herbivorous ant samples, compared to just 5% of omnivore OTUs (17% of samples) and 1% of predator OTUs (5% of samples) (Fig. S2a). Almost two-thirds of Enterobacterales OTUs in herbivores could not be assigned to a family, while the rest belonged to *Enterobacteriaceae* but were unclassified beyond that, indicating notable 16S rRNA divergence from free-living relatives. This includes uncharacterized reads in the obligate herbivore and ant-plant mutualist *Philidris nagasau* and the omnivore *Overbeckia papuana*. BLAST results indicated that the bacteria in *P. nagasau* were related to a *Sodalis*-allied bacteria (>94% similarity), while the bacteria in *O. papuana* were the endosymbiont *Blochmannia,* as would be expected for an ant species in the Camponotini tribe (Table S2). Two additional unclassified OTUs from *Anonychomyrma minuta* and *Crematogaster borneensis* aligned closely with the tsetse fly secondary endosymbiont *Sodalis glossidanus*. The genus *Arsenophonus*, which is a common intracellular symbiont of insects, was also found in high abundance in the omnivorous *Technomyrmex brunneus*.

Finally, the orders Entomoplasmatales and Burkholderiales also revealed interesting patterns. Entomoplasmatales was most common in herbivores but found across all feeding groups and consistently represented by two families: Entomoplasmataceae and Spiroplasmataceae; it was especially abundant in *Vombisidris* sp*.* 4. Burkholderiales was strongly associated with invasive *Anoplolepis gracilipes*, the “yellow crazy ant,” where it dominated the microbiome: these were identified only to the family level, *Oxalobacteraceae*.

### Differences between developmental stages

Although sample sizes were insufficient for statistical analysis, developmental and caste-based bacterial community shifts were observed in several species. In *Vombisidris*, early stages appear to be dominated by Enterobacterales and *Wolbachia*, while adults harbored Entomoplasmatales that were absent in earlier stages (Fig. S2b). In *Pheidole hospes*, Flavobacteriales appeared only in workers, presenting at low abundance in pupae but were absent in queens and larvae (Fig. S2b). Interestingly, *Strumigenys* workers in both species have a highly structured bacterial community consisting of the orders Sphingomondales, Propionibacteriales, Sphingobacteriales, Pseudocardiales, Rhizobiales, and Rickettsiales as adults; in larvae, we observed only Rickettsiales and low levels of Flavobacteriales and Pseudomonadales.

Our results suggest that Rhizobiales is commonly acquired during later developmental stages in predatory and omnivorous ants (Fig. S2b). In the predator *Solenopsis papuana*, workers and a single pupa sampled consistently harbor Rhizobiales, but larvae are entirely dominated by *Wolbachia*. A similar trend may also be present in *Rhytidoponera* cf. *strigosa*, where Rhizobiales dominated adult microbiomes but was not present in the single larvae sampled. In the omnivore *Tetramorium* sp. 6 cf. *validiusculum*, Rhizobiales appeared only in adults but were absent in larvae and pupae. Finally, the previously mentioned highly structured bacterial community of the *Strumigenys* genus, which also includes Rhizobiales, does not appear until adulthood either.

### Microbial acquisitions linked to the evolution of host ecological traits

Using BayesTraits V4 (41), we found strong support for an association between Enterobacterales and the evolution of herbivory (logBayesFactor = 45; Table 1), suggesting that transitions to herbivorous diets in ants coincide with increased Enterobacterales abundance (Fig. 4). A weaker correlation was also found between Rickettsiales (*Wolbachia*) and higher δ^15^N values (logBayesFactor = 11). No significant correlation was identified between Rhizobiales and the evolution of diets across the ant phylogeny. These results should be interpreted with caution due to the relatively small sample size of herbivorous and predatory lineages in our data set.

**TABLE 1:** BayesTraits results testing correlations between bacterial order acquisition and ant feeding niche measured by δ^15^N^*a*^

Metric	Bayes continuous
	δ^15^N mean ~ mean (abundance)
Enterobacterales	45
Rickettsiales	11
Rhizobiales	<1

^*a*^ Log Bayes Factors (logBF) >2 indicate a positive association; >10 indicate strong support. Values are rounded to whole numbers; very small values are shown as <1.

## DISCUSSION

Our results revealed substantial variation in the microbiomes of Indo-Pacific ants, with patterns both supporting and contradicting those observed in Neotropical lineages. Bacterial community diversity and composition were strongly influenced by dietary protein levels, with more predaceous ants hosting higher bacterial diversity. We also found associations between specific bacterial orders and the evolution of feeding ecologies, such as Enterobacterales and the transition to herbivory in ants. Additionally, data suggests bacterial community composition may vary across developmental stages and castes in some species.

### Rhizobiales are frequently associated with higher protein diets in Indo-Pacific ants

Strong associations with Rhizobiales were found in 5 of 8 predatory ant species but were completely absent in strict herbivores (δ^15^N < 2). This contrasts with previous studies where the presence of Rhizobiales was linked to the evolution of herbivory in ants (12). In particular, higher scoring δ^15^N species *Aenictus huonicus* and *Rhytidoponera* cf. *strigosa* showed individuals with Rhizobiales abundance as high as 99%. Interestingly, the OTUs in *A. huonicus* closely matched *Candidatus Tokpelaia hoelldoblerii*, a symbiont in the predatory *Harpegnathos saltator* that may aid in nitrogen recycling (21). Predacious *Solenopsis papuana* also carried Rhizobiales at >50% relative abundance on average across individuals, suggesting a close host-microbe relationship, with the remainder composed of *Wolbachia*, commonly found in endemic *Solenopsis* populations (42). *Bartonella*-like Rhizobiales are also found in omnivorous giant bullet ants, *Paraponera clavata* (43, 44). The Rhizobiales occurrence we found in omnivorous *Tetramorium validiusculum* and *Tetraponera laeviceps* further supports their widespread presence in these genera (12). The prevalence of Rhizobiales—typically linked to nitrogen fixation and herbivory—in predatory ants consuming nitrogen-rich diets was unexpected. Our findings identified at least three new predacious lineages with strong Rhizobiales associations, suggesting nutrient recycling or provisioning via Rhizobiales may be more common in predatory ants than previously thought. Comparing Rhizobiales genomes in these ants could reveal convergent metabolic signatures that may shed light on their functional role in predatory ant microbiomes.

### Predatory ants tended to host a greater diversity of microbes

We found that ants with higher δ^15^N scores, indicating a more protein-rich diet, were associated with high bacterial diversity. Omnivorous insects tend to host more diverse gut microbiota due to their varied diets, compared to herbivores or specialist predators such as ladybird beetles (45, 46). Most predatory ants in our data set are generalists feeding on a range of arthropods, likely contributing to their increase in bacterial diversity, particularly in *Strumigenys* and *Brachyponera*.

Adults of the *Strumigenys* species we investigated appeared to have structured microbiomes consisting of six dominant bacterial orders at consistent relative abundances across individuals (Fig. S2a). These included common insect-associated taxa like Rhizobiales and Rickettsiales, as well as Pseudonocardiales and Sphingobacteriales, which are thought to play beneficial roles in antimicrobial production and nitrogen waste recycling in fungal-growing ants and leeches, respectively (27, 47–49). In predatory *Brachyoponera croceicornis*, Pseudomonadales dominated, but Micrococcales and Flavobacteriales were also present. Stable microbiomes have been previously reported in other predatory ants, such as the Argentine ant (*Linepithema humile*), which maintains *Lactobacillus*, *Acetobacteraceae*, and *Rickettsia* regardless of diet or geography (31), and the trap-jaw ant (*Daceton armigerum*), which consistently harbors Rhizobiales and Entomoplasmatales (28). While it is well established that Rhizobiales occurs across various ant species, its presence has generally been less common in predatory ants; for instance, *Daceton* is a notable exception, and our findings add to this pattern.

Several omnivorous species, such as *Philidris cordata*, also showed increased microbial diversity aside from *Wolbachia*, while *Crematogaster polita* and *Parapratrechina* sp*.* 10 had inconsistent microbiomes, suggesting transient associations. Conversely, the omnivorous yellow crazy ant (*Anoplolepis gracilipes*) exhibited low diversity, with the genus *Burkholderia* dominating. *Burkholderia* includes gut-associated insect symbionts, such as in bean bugs and *Cephalotus* turtle ants, involved in nitrogen recycling (15, 50, 51). Bacterial diversity was lowest in herbivorous ants, which averaged only four bacterial orders. This pattern mirrors broader insect trends, where herbivores often possess simplified, specialized gut-associated microbiota, or obligate symbionts, dominated by one or few taxa (46, 52).

### Enterobacterales acquisitions correlate with the evolution of herbivory

Analysis of correlated evolution suggested that Enterobacterales acquisition has repeatedly accompanied the evolutionary transition toward herbivory in ants. This pattern is also supported by dietary analysis showing these bacteria are more prevalent in species with lower protein intake (Fig. 4). Enterobacterales are known for forming obligate nutritional mutualisms with various insect lineages, often coinciding with nutrient-imbalanced herbivorous diets (3, 12, 53). At least four ant lineages—*Formica*, *Cardiocondyla*, *Plagiolepis*, and the Camponotini tribe—have independently evolved obligate symbioses with Enterobacterales (8). In these relationships, the bacteria reside in specialized host cells called bacteriocytes and are strictly passed down to offspring (8, 20, 54). In *Camponotus*, the symbiont *Blochmannia* enhances colony fitness on amino acid-poor diets by aiding in nutritional upgrading (20). Genomic evidence suggests similar roles for symbionts in *Formica*, *Plagiolepis*, and *Cardiocondyla*, including provisioning of essential amino acids like tyrosine, which is essential for cuticle formation but often lacking in low-protein diets (8, 54).

We identify Enterobacterales-derived bacteria in *Philidris nagasau* and *Overbeckia papuana* sp., with OTUs showing deep divergence from free-living relatives. BLAST results indicate both microbes are related to *Sodalis*-allied symbionts. Our results showed evidence that *Overbeckia*, a Camponotini member, indeed harbors *Blochmannia* as speculated in Klimes et al. (55) while *Philidris nagasau*, an herbivore and ant-plant mutualist, contains an uncharacterized *Sodalis*-like bacterium. Interestingly, Enterobacterales are also common in ant-plant mutualists *Anonychomyrma minuta* and *Crematogaster borneensis*, suggesting associations of these ant microbes with herbivory—and possibly plant mutualisms—may be more widespread than previously recognized.

We also detected Enterobacterales in only one of the two herbivorous trophobiont-tending ant species, *Technomyrmex brunneus*, with this bacterial order otherwise absent from the group. Feeding on honeydew from sap-feeding insects is usually associated with a more herbivorous diet in ants. We may therefore expect these ants to follow trends seen in herbivores, and to some extent, they do show simpler microbiomes (Fig. S2c). However, some species also exhibit patterns more typical of predators, such as *Rhizobiales* associations in *Tetraponera laeviceps* and *Crematogaster modigliani*, and the complex bacterial communities in *Crematogaster polita* and *Paraparatrechina* sp. 10. We also found no evidence of *Lactobacillus* in trophobiont-tending ants, which had been previously reported (56), although it may be present in a broader sample of Indo-Pacific ant lineages.

Through our evolutionary analysis, we also found a weak correlation between *Wolbachia* and species with more protein-rich diets, particularly omnivores, although this correlation explained limited variance in our linear modeling (Fig. 4). In our data set, *Wolbachia* dominated the bacterial community of several species across all feeding guilds, and its dynamics across ant lineages was highly variable. Studies on *Monomorium* and *Tapinoma* ants have suggested *Wolbachia* may provide fitness benefits, while others show its absence in invasive *Solenopsis* populations despite thriving in native ones (10, 42, 57–59). Within-host *Wolbachia* density can be influenced by diet in other insects, such as *Drosophila* (60). To our knowledge, our study is the first to show a link between diet and the incidence of *Wolbachia* infection in ants; however, this should be viewed with caution due to our relatively low phylogenetic sampling of the Formicidae. Future research should investigate how herbivorous and predatory diets or lifestyles affect *Wolbachia* infection to identify factors that may promote or suppress its presence.

### Ant microbiomes can differ across developmental stages and castes

Although sampling across developmental stages is limited, our data suggest that some ant species exhibit distinct microbiomes between early developmental stages and adulthood (Fig. S2b). In particular, Rhizobiales showed a strong correlation with dietary protein levels, but this only appears to emerge in later developmental stages, being primarily present in adult workers of predatory *S. szalayi*, *Solenopsis papuana*, and *Rhytidoponera cf. strigosa*, with a more stable, structured bacterial community only becoming fully apparent in *S. szalayi* adults. Similarly, in herbivorous *Vombisidris* sp*.* 4*,* we only see the association with Entomoplasmatales, usually found in predatory army ants, in adult workers (25, 61, 62). These patterns suggest that adult microbiomes may develop during or after metamorphosis in some Indo-Pacific ant species. Prior research shows bacterial load and composition can vary across developmental stages (17, 28, 63). For example, in *Diacamma cf. indicum,* Firmicutes are exclusive to adult worker guts, a pattern also observed in *Eciton* and *Labidus* army ants, although the function is unclear in workers (64). These findings indicate that microbiota may serve different functions across different life stages, and that Indo-Pacific ants may provide a valuable system for studying caste- and development-dependent microbiomes.

### Nesting habitat does not influence ant microbiota

We found no evidence that nesting habitat influenced ant bacterial community diversity (Fig. S2d). This contrasts with previous work suggesting that nesting mode can influence ant microbiomes (29). However, our focus was only on arboreal and ground-nesting species. Future studies investigating specialized nesting habitats and more biomes could clarify whether these behaviors correlate with differing microbial associates.

### Conclusion

Our results both support and refine previous understandings of the associations between certain bacteria and feeding ecology in ants, highlighting areas of consistency as well as important differences. We also uncovered new relationships between predatory and herbivorous ant lineages and their microbiota: predatory ants host greater bacterial community diversity and show surprisingly frequent associations with Rhizobiales, and we found evidence supporting the acquisition of Enterobacterales accompanying the evolutionary transition toward herbivory in ants. Our findings provide valuable insights into the diversity and evolution of ant-microbe interactions, underscoring the importance of Indo-Pacific ants as a promising system for expanding current theories on bacterial associations linked to specialized lifestyles.

## MATERIALS AND METHODS

### Sample collection and design

In total, we sequenced 267 individual ants from several castes and life stages across 36 species, 24 genera, and 7 subfamilies (mostly workers, see Results for details). Of these, 31 species were collected from Papua New Guinea, representing the most common genera and/or species sampled in trees in lowland and mid-elevation forests (65, 66) (Table S1). The remaining species were collected from Malaysian Borneo and Fiji. All samples were collected using the protocol laid out in (67). The final data set included 5 herbivorous, 24 omnivorous, and 7 predaceous species, as well as 4 ant-plant mutualists, 15 trophobiont-tending mutualists, and 17 species presenting no observed mutualism with plants or trophobionts. As this collection included sampling from nests, we were also able to analyze queens and larvae for some of the species (Fig. S2a through d). Ants were stored in 96% ethanol in a −20°C freezer. Details on all samples and collection sites, including GPS location, can be found in Table S1.

### Data collection and processing of bacterial reads

DNA extractions were performed on whole bodies using the Qiagen DNeasy Blood & Tissue kit (QIAGEN Ltd., Manchester, United Kingdom), following the manufacturer’s protocol. A single ant was used for each sample and was first washed of surface microbes using sterile water to ensure that mostly the internal bacterial community was sequenced and then crushed using a pestle before commencing the extraction. To sequence the bacterial community present in the samples, we amplified the highly conserved V4 region of the 16S rRNA gene using tagged Caporaso primers: 515F 5′ GTGCCAGCMGCCGCGGTAA′3 and 806R 5′ GGACTACHVGGGTWTCTAAT ′3 (68). PCR reactions consisted of 12.5 µL Q5 High Fidelity 2× Master Mix, 0.5 µL of each primer, and 9.5 µL sterile H_2_O, with 2 µL of DNA per reaction. PCR conditions used were initial denaturation at 98°C (30 s), then 25 cycles of 98°C (10 s), 50°C (15 s), 72°C (20 s), and final extension step of 72°C (5 min). PCR products were then imaged using gel electrophoresis to confirm size and concentration and then sent to the Liverpool Genome Center for purification, addition of indices and adapters, and sequenced on one Illumina MiSeq v2 run at a depth of 12 million paired-end reads, with additional PhiX to increase library diversity. Two negative controls were used for each sequencing plate, one using a sample generated from a complete DNA extraction without an ant to control for the DNA extraction kit and one a PCR reaction without a DNA template to control for the PCR mix; this represented 12 negative controls in total.

### Processing of amplicons

The demultiplexed Illumina sequencing reads were filtered and processed using the well-documented DADA2 denoising pipeline from QIIME 2 2024.5 (69, 70). Taxonomic classification of the 16S Amplicon Sequence Variants (ASVs) was performed using the command “classify-sklearn” with SILVA reference database version 138 (71, 72) provided by QIIME2. The reads were then cleaned of mitochondria, chloroplasts, and archaea, as well as microbial contaminants identified and removed using the “prevalence” method of the decontam package in R (73). The ASVs were then clustered into OTUs at 99% similarity, which were used for all analyses. Using the rarefaction curve generated in QIIME2, we assigned 1,000 reads per sample as our minimum frequency to characterize the bacterial community. Samples with <1,000 reads were removed from the analysis, and only OTUs with an abundance of greater than 1 read count were included, resulting in 239 samples being analyzed (Table S3). All code and pipelines created and followed for this section are available in the Supplementary material.

### Characterization of bacterial community diversity

Diversity analyses were conducted in QIIME2, including reconstruction of 16S rRNA bacterial phylogenies. Bacterial alpha-diversity was assessed using Shannon’s entropy (species richness and evenness) and Faith’s PD (richness incorporating phylogenetic relatedness, without abundance weighting). Results were visualized as scatterplots in ggplot2 (74).

We also characterized bacterial community composition between samples using Bray-Curtis, Jaccard, weighted-UniFrac, and unweighted-UniFrac metrics, to account for the influences of relative abundance (e.g., Bray-Curtis) and bacterial phylogeny (e.g., UniFrac) on community composition (29). All distance matrices were visualized using principal coordinate analysis (PCoA); this was preferred over non-metric multidimensional scaling as it provides fixed results (e.g., PC1 eigenvalue) that can be used in downstream statistical analysis and visualization of patterns. PCoA results were generated using the command “pcoa” from the R package ape (75) and plotted using “geom_point” and “geom_polygon” from the package ggplot2 (74). All diversity data can be found in Table S4.

### Ecological traits

To characterize ant species diets, we analyzed newly generated and previously published natural abundance stable isotope nitrogen data, which reflects dietary position: higher δ^15^N values indicate predatory or scavenging diets, while lower values suggest plant-based diets (76). The δ^15^N values represent the ratio of heavy nitrogen (^15^N) to light nitrogen (^14^N) in ant tissues relative to a reference standard (77, 78). For most species, ethanol-preserved ant tissues were analyzed using stable isotope ratio mass spectrometry. Samples consisted of pooled tissues from approximately 2–20 workers per colony (depending on body size), with abdomens removed to avoid gut-content bias. Tissues were dried overnight at 60°C, ground into powder, and 0.1–1.1 mg dry weight was packed in silver foil capsules for mass spectrometry at Boston University’s Stable Isotope Laboratory. The isotope values were baseline-corrected using either plant tissue or topsoil means from collection sites (depending on the sampled stratum), and then the species means were calculated. For seven species lacking direct data, we either estimated δ^15^N using our own measurements from closely related species or used published genus-level data (39, 79, 80) (Table S1).

For categorical analyses, we classified species into three dietary guilds—herbivore, omnivore, and predator—based solely on the stable isotope data, using the following δ^15^N thresholds: 0–2 for herbivores, 2–6 for omnivores, and 6–8 for predators (Table S2). We based these thresholds on previous literature (12, 81), finding they broadly align with published diet classification (82, 83), and they were only used for visualization and SIMPER analysis (outlined below). Nesting habitat was categorized as arboreal (tree-nesting) or ground-nesting (soil/underground), based on our field observations and literature sources (55, 79, 80, 82, 84). We also recorded mutualism type (ant-plant, trophobiont-tending, or no mutualism) but excluded it from statistical analyses due to strong correlation with the diet (Fig. S5). Full details are available in Tables S1 and S2, and bacterial community trends across mutualism type in Fig. S2c.

### Host housekeeping genes and host phylogenetic reconstruction

The host ant phylogeny was generated using the Cytochrome Oxidase I and Wingless genes. Sequences were obtained from NCBI and published phylogenies, supplemented by our own sequencing of Wingless for underrepresented taxa (see Table S5). In-house sequencing followed the protocols from Larabee et al. (85) using primers Wg550F 5′ ATGCGTCAGGARTGYCAYGGYATGTC ′3 and Wg1023R 5′ ACYTCGCAGCACCARTGGAA ′3 with the PCR reactions conditions: 94°C (5 min), followed by 10 cycles of 94°C (30 s), 60°C (30 s, decreasing by 1°C per cycle), and 72°C (30 s), followed by 30 cycles with the same conditions but an annealing temperature of 50°C, and a final extension of 72°C (5 min) (85). Amplicons were sequenced at Eurofins (Guildford, UK).

The tree alignment was built in Geneious Prime 2024.0.5 and exported as a NEXUS file. Constraints were applied based on Hoenle et al. (65) to account for the limited deep-node resolution of fast-evolving genes. Phylogenies were inferred using MrBayes v5.3.0 with two runs of 50 million generations (5,000 iteration checkpoints) and a mixed + gamma substitution model (65, 86, 87). The consensus tree was visualized in FigTree 1.4.4, imported into R for analysis, and pruned for study species using the “drop.tip” function from the ape package (75). The full tree is available in Fig. S1. The reduced tree was edited in Inkscape 1.2.2 for visualization. Sequence details are in Table S5, and NEXUS tree and alignment files can be found in the Supplementary material.

### Statistical analyses

We tested whether the ecological traits (diet and nesting habitat) correlated with bacterial diversity using generalized linear mixed modeling with Markov Chain Monte Carlo methods using the R package MCMCglmm (88), with host ant phylogeny included as a random effect. For community composition, we used the eigenvalues from principal coordinate one (PC1) from each principal coordinate analysis of each metric (Bray-Curtis, Jaccard, etc.) as the response variable with each trait individually in the structure “PC1 ~ Habitat/d^15^N.” Alpha-diversity was modeled in the same way using the logged Shannon and Faith’s PD vectors generated in QIIME 2.

### Contribution of specific bacteria to bacterial community diversity

To evaluate the contribution of specific bacteria to ant bacterial community diversity, we relied on SIMPER tests from the R package vegan (89), which assesses which bacteria were contributing the most to the dissimilarity observed between different categorical diet groups (based on stable isotope data) (90). Linear modeling using the function “lm” from package lme4 (91) was also used to test the correlation between the top three bacterial orders that most contributed to the variance observed—Rickettsiales, Rhizobiales, and Enterobacterales (as discovered through SIMPER analysis)—and the d^15^N values per species, these were each performed on subsets filtering for each order individually, i.e., “Enterobacterales_abundance ~ d^15^N.”

### Phylogenetic correlation of ant feeding niche and bacterial orders

We also asked if the evolution of particular ecological traits is associated with the increased relative abundance of certain bacteria using BayesTraits V4 (41, 92) using our host phylogenetic tree. We only tested correlations with the top three bacterial orders most influencing the variance observed between groups. We used Bayes Continuous to test for evolutionary correlations between diet, based on stable isotope data, and the relative abundance of Rickettsiales, Rhizobiales, and Enterobacterales across species in the ant phylogeny. We ran two models—one assuming correlation and one assuming none—then calculated the log Bayes Factor (BF) using Log BF = 2(log marginal likelihood complex model – log marginal likelihood simple model). A log BF of 2–6 indicates correlation, 6–10 suggests strong correlation, and >10 is considered very strong (93).

## Data Availability

All data presented in this study can be found online at figshare (https://doi.org/10.6084/m9.figshare.29490968) and NCBI (BioProject PRJNA1290175, SRX29699679:SRX29699988).

## SUPPLEMENTAL MATERIAL

Supplemental material spectrum.01965-25-s0001.docx Tables S6 and S7; Fig. S1 to S5.

Table S1 spectrum.01965-25-s0002.xls Metadata on all samples.

Table S2 spectrum.01965-25-s0003.xls Metadata on individual species.

Table S3 spectrum.01965-25-s0004.xls Details on OTUs, taxonomic and sample origin.

Table S4 spectrum.01965-25-s0005.xls Data from diversity analyses.

Table S5 spectrum.01965-25-s0006.xls Details on gene sequences used to create phylogeny.

Table S8 spectrum.01965-25-s0007.xls All results from SIMPER analyses, many quoted in the main text.

Table S9 spectrum.01965-25-s0008.xls Summaries across OTUs and samples to get overview of bacterial presence/absence, stats from here quoted in the main text.

ASM does not own the copyrights to Supplemental Material that may be linked to, or accessed through, an article. The authors have granted ASM a non-exclusive, world-wide license to publish the Supplemental Material files. Please contact the corresponding author directly for reuse.
