# Supplementary material for: Dietary niche shapes bacterial community in Indo-Pacific ants
Source: Microbiol Spectr. 2025 Sep 3;13(10):e01965-25. doi: 10.1128/spectrum.01965-25 (PMC12502676; doi:10.1128/spectrum.01965-25)
Supplement: Supplemental material — Tables S6 and S7; Fig. S1 to S5. [file spectrum.01965-25-s0001.docx]

| α-diversity metric | | Model outputs | | | |  |
| --- | --- | --- | --- | --- | --- | --- |
| Faith’s PD | | *Posterior mean (β)* | *95% CI (lower, upper)* | *% > 0* | *pMCMC* | *DIC* |
| *Stable isotope* | **δN15** | ***0.20*** | ***(0.08, 0.32)*** | ***>99%*** | ***0.002 ***** | **364** |
| *Nesting mode* | Arboreal (intercept)  Ground | 2.11  -0.11 | (0.52, 3.64)  (-0.69, 0.48) | -  36% | -  0.71 | 363 |
| *Stable isotope + Nesting Mode* | Arboreal (intercept)  **δN15**  δN15 + Ground | 1.14  **0.24**  -0.42 | (-0.25, 2.52)  **(0.11, 0.37)**  (-0.98, 0.09) | -  **>99%**  6% | -  **0.001 ****  0.11 | **363** |
| Shannon | | | | | | |
| *Stable isotope* | **δN15** | **0.32** | **(0.15, 0.50)** | **>99%** | **0.001 **** | **659** |
| *Nesting mode* | Arboreal (intercept)  Ground | -0.43  -0.18 | (-2.64, 1.81)  (-1.08, 0.73) | -  35% | -  0.71 | 658 |
| *Stable isotope + Nesting Mode* | Arboreal (intercept)  **δN15**  δN15 + Ground | -1.97  **0.38**  -0.70 | (-3.89, -0.02)  **(0.20, 0.57)**  (-1.53, 0.10) | -  **>99%**  5% | -  **0.0002 ****  0.09 | **657** |

** p value – significant (<0.05) and close to significant (<0.06) results are shown in* ***bold***

**Table S6. Model output from MCMCglmm of alpha diversity with diet and nesting mode.** Significance is shown by a posterior mean above or below zero, and credible intervals (CI) that do not include zero, if these criteria are met then a significant p value can be accepted and there is strong certainty the effect is significant. Host phylogeny is included as a random effect.

| Community composition metric (PC1) | | Model outputs | | | |  |
| --- | --- | --- | --- | --- | --- | --- |
| Jaccard | | *Posterior mean (β)* | *95% CI (lower, upper)* | *% > 0* | *pMCMC* | *DIC* |
| *Stable isotope* | **δN15** | **-0.04** | **(-0.06, -0.02)** | **<1%** | **0.002 **** | **-480** |
| *Nesting mode* | Arboreal (intercept)  Ground | -0.01  -0.01 | (-0.30, 0.29)  (-0.12, 0.10) | -  44% | -  0.87 | -481 |
| *Stable isotope + Nesting Mode* | Arboreal (intercept)  **δN15**  δN15 + Ground | 0.17  **-0.04**  0.05 | (-0.11, 0.42)  **(-0.07, -0.02)**  (-0.05, 0.15) | -  **<1%**  83% | -  **0.001 ****  0.35 | **-480** |
| Bray-Curtis | | | | | | |
| *Stable isotope* | δN15 | -0.04 | (-0.09, 0.02) | 9% | 0.18 | -264 |
| *Nesting mode* | Arboreal (intercept)  Ground | -0.02  -0.12 | (-0.63, 0.62)  (-0.35, 0.10) | -  13% | -  0.26 | -265 |
| *Stable isotope + Nesting Mode* | Arboreal (intercept)  δN15  δN15 + Ground | 0.11  0.03  -0.08 | (-0.57, 0.77)  (-0.09, 0.03)  (-0.32, 0.15) | -  15%  24% | -  0.40  0.39 | -264 |
| Weighted unifrac | | | | | | |
| *Stable isotope* | δN15 | -0.01 | (-0.04, 0.02) | 35% | 0.70 | -671 |
| *Nesting mode* | Arboreal (intercept)  Ground | 0.00  -0.06 | (-0.33, 0.35)  (-0.17, 0.06) | -  16% | -  0.31 | -671 |
| *Stable isotope + Nesting Mode* | Arboreal (intercept)  δN15  δN15 + Ground | 0.01  0.00  -0.06 | (-0.34, 0.38)  (-0.03, 0.03)  (-0.17, 0.06) | -  49%  18% | -  0.97  0.35 | -671 |
| Unweighted unifrac | | | | | | |
| *Stable isotope* | **δN15** | **0.05** | **(0.02, 0.09)** | **>99%** | **0.003 **** | **-232** |
| *Nesting mode* | Arboreal (intercept)  Ground | -0.02  -0.04 | (-0.45, 0.40)  (-0.19, 0.13) | -  33% | -  0.66 | -234 |
| *Stable isotope + Nesting Mode* | Arboreal (intercept)  **δN15**  δN15 + Ground | -0.30  **0.07**  -0.12 | (-0.67, 0.08)  **(0.03, 0.10)**  (-0.27, 0.02) | -  **>99%**  5% | -  **0.001 ****  0.1 | **-233** |

** p value – significant (<0.05) and close to significant (<0.06) results are shown in* ***bold***

**Table S7. Model output from MCMCglmm of community composition with diet and nesting mode.** Significance is shown by a posterior mean above or below zero, and credible intervals (CI) that do not include zero, if these criteria are met then a significant p value can be accepted and there is strong certainty the effect is significant. Host phylogeny is included as a random effect and principle coordinate 1 (PC1) was used for each community composition metric.

**Figure S1. Full phylogeny of ants based on Cytochrome Oxidase I and Wingless gene sequences.** Backbone alignment taken from Hoenle et al 2023 tree with added support from Moreau et al 2006, NCBI and our own wingless sequences. The alignment was built and refined in Geneious Prime 2024.0.5 and then exported as a NEXUS file. Constraints at a subfamily and tribe level were added to the NEXUS file and the tree generated using MrBayes v5.3.0 with two runs of 50 million generations and checkpoints every 5,000 iterations. For each partition we applied the nucleotide substitution scheme ‘mixed + gamma’. The consensus tree from Mr Bayes was then visualised in FigTree 1.4.4. The tree alignment and NEXUS file can be found in online Supplementary Material.

1. Bacterial community composition by feeding guild (assigned by δ^15^N scores)

(b) Bacterial community composition by life stage, caste and feeding guild (δ^15^N score)

(c) Bacterial community composition by mutualism type

(d) Bacterial community composition by nesting habitat

**Figure S2. Taxonomic bar plots showing overview of individual ant microbiomes across species in our study.** The bar plots are split into (a) feeding guild (herbivore, omnivore, predator) based on δ^15^N scores; (b) life stage, caste and feeding guild (only for species where these samples were available); (c) mutualism type (ant-plant mutualists, trophobiont-tending mutualists and no observed mutualism) and (d) nesting habitat (arboreal or ground-dwelling). Relative abundance is shown on the y axis and species name on the x axis. Colours denote the bacterial order present (split colours refer to multiple OTU’s within the same order), orders present at <1% are grouped in grey as “Other”. Some species are represented by multiple colonies.


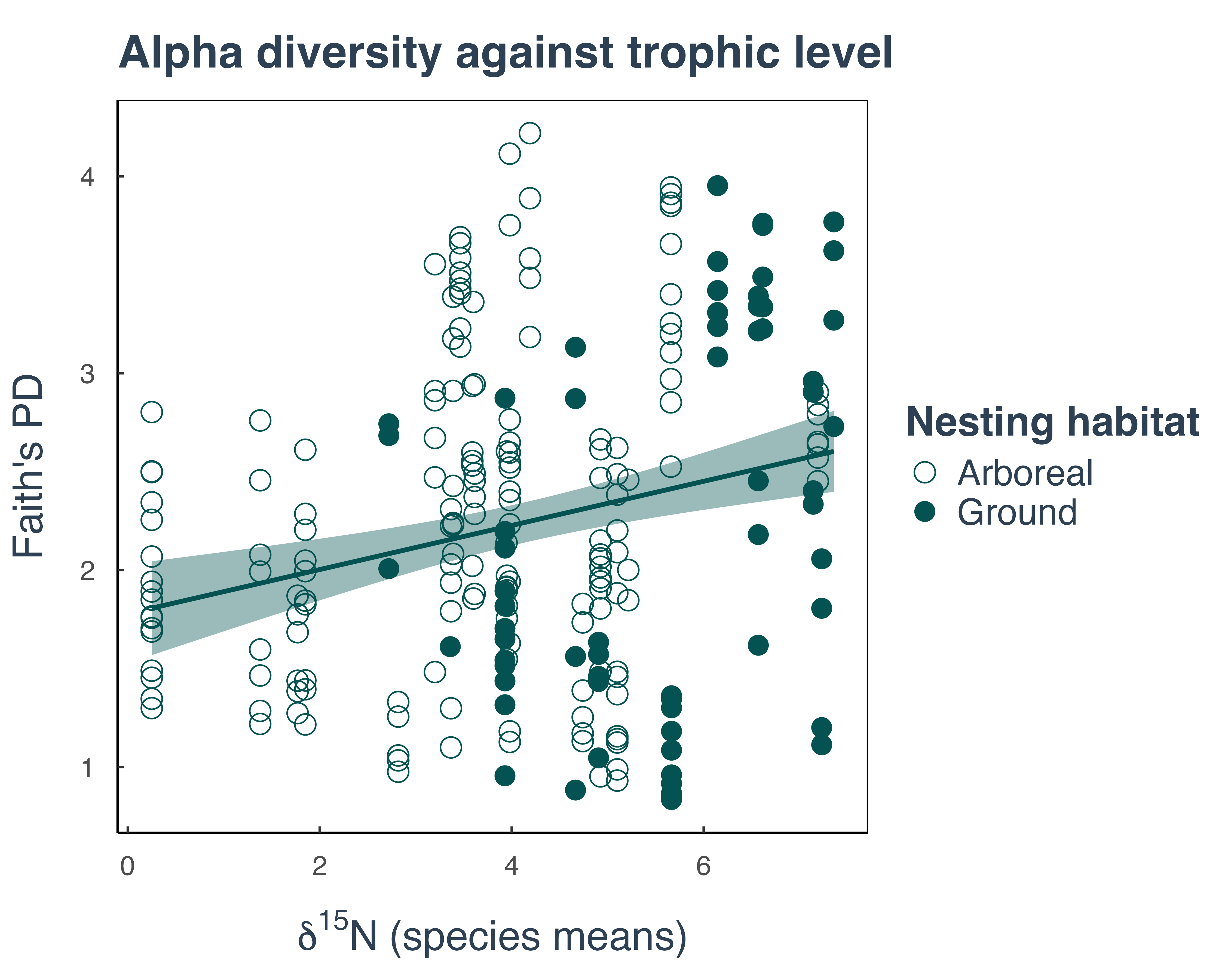


**Figure S3. Scatter plot showing alpha diversity with δ^15^N.** Alpha diversity represented by Faith’s PD shows a positive correlation with the amount of protein in a species diet. Points were generated from mean δ^15^N score per species, filled shapes indicate arboreally nesting species and unfilled points represent ground-nesting species. Statistics were generated from MCMCglmm models, the full outputs are shown in supplementary Table S3.

**

**

**Figure S4. Principle coordinate analysis plots of bacterial community composition.** Community composition represented by Bray-Curtis and Weighted UniFrac, coloured by (a-b) feeding guild and (c-d) nesting habitat. Significance values for MCMCglmm models of community composition with functional traits can be found in Supplementary Table 6.


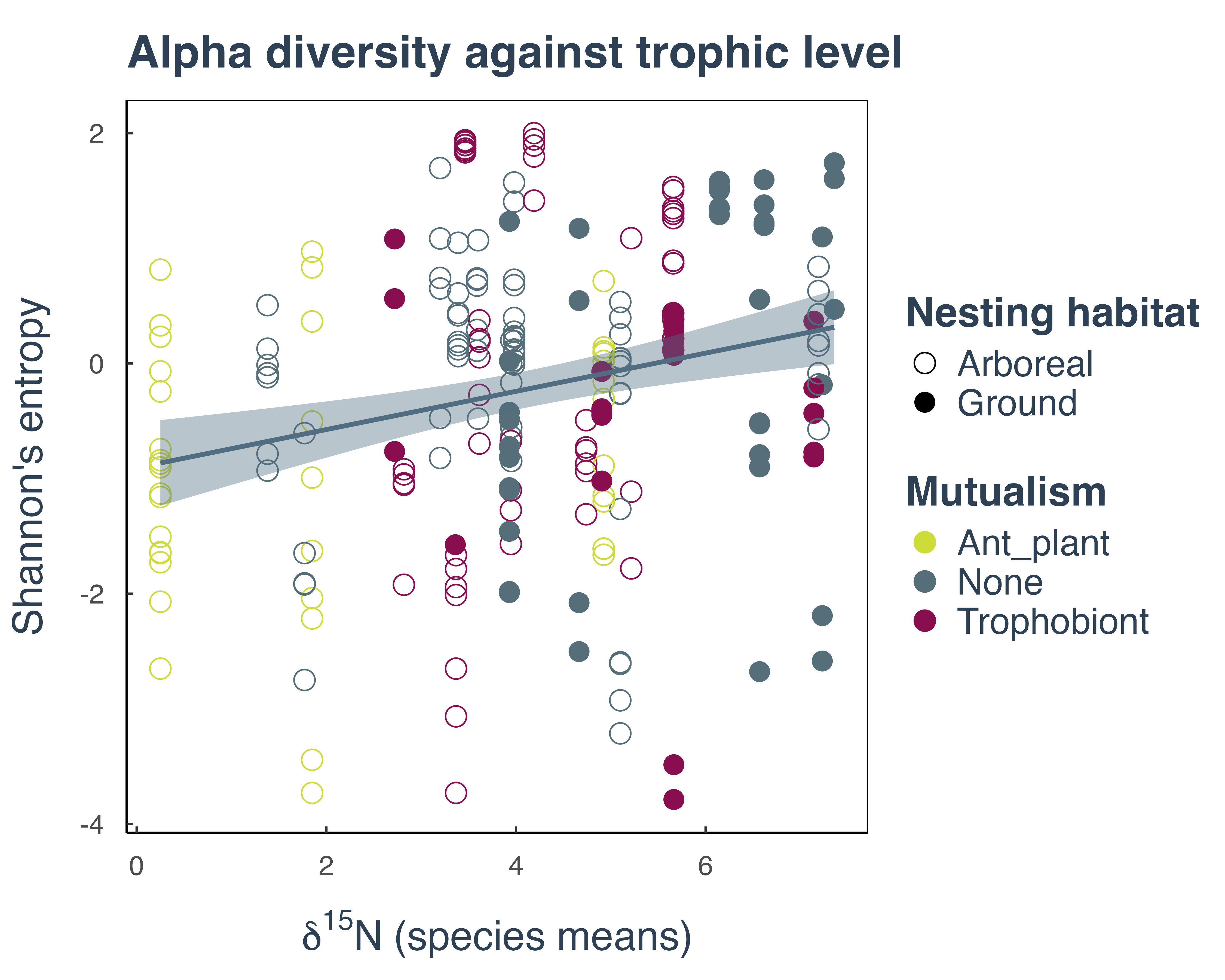


**Figure S5. Ecological trait correlations.** Alpha diversity represented by Shannon’s entropy shows a positive correlation with the amount of protein in a species diet. Points are coloured by mutualism type and filled or unfilled based on nesting habitat to demonstrate inherent correlation between traits. This was statistically tested using linear modelling: δ^15^N ~ mutualism_type (Adjusted R-squared: 0.1788

F-statistic: 4.81, 33 DF, p-value: 0.01468) and δ^15^N ~ nesting_habitat (Adjusted R-squared: 0.2513, F-statistic: 12.75, 34 DF, p-value: 0.002).
